# Sophocarpine inhibits tumor progression by antagonizing the PI3K/AKT/mTOR signaling pathway in castration-resistant prostate cancer

**DOI:** 10.7717/peerj.14042

**Published:** 2022-09-16

**Authors:** Min Weng, Chenghao Shi, Hui Han, Hengyue Zhu, Yanyi Xiao, Hangcheng Guo, Zhixian Yu, Cunzao Wu

**Affiliations:** 1 Department of Urology, The First Affiliated Hospital of Wenzhou Medical University, Wenzhou, Zhejiang, China; 2 Key Laboratory of Diagnosis and Treatment of Severe Hepato-Pancreatic Diseases of Zhejiang Province, The First Affiliated Hospital of Wenzhou Medical University, Wenzhou, Zhejiang, China

**Keywords:** Castration-resistant prostate cancer, Sophocarpine, mTOR, Proliferation, Epithelial-mesenchymal transition

## Abstract

**Objective:**

The objective of this study was to investigate the inhibitory effect of sophocarpine on the progression of castration-resistant prostate cancer (CRPC) and the underlying molecular mechanism.

**Methods:**

DU145 and PC3 cells (two CRPC cell lines), incubated with different concentrations of sophocarpine, were used. Cell Counting Kit-8 assay, real-time cellular analysis, and colony formation assay were conducted to evaluate the proliferation of CRPC cells. Cytometry flow analysis was performed to evaluate the apoptosis rate of CRPC cells. Wound healing and Transwell invasion assays were performed and the levels of the epithelial-mesenchymal transition (EMT)-related proteins were determined to analyze cell migration and invasion abilities. A xenografted tumor model of nude mice was used to examine the anti-cancer effect of sophocarpine on CRPC. Western blotting was performed to evaluate the activities of the PI3K/AKT/mTOR signaling pathway both in cells and tumor tissues.

**Results:**

*In vitro* tests showed that sophocarpine suppressed the proliferation of CRPC cells, reduced the migration and invasion abilities, and increased the apoptosis rate. *In vivo*, sophocarpine decreased the weight and volume of tumor tissues. Mechanically, sophocarpine exerted its anti-cancer effects by inactivating PI3K/AKT/mTOR signaling.

**Conclusion:**

Sophocarpine inhibited the progression of CRPC by downregulating the PI3K/AKT/mTOR signaling pathway and showed a potential to be an anti-cancer agent against CRPC.

## Introduction

Prostate cancer has a high prevalence globally (Siegel, Miller & Jemal, 2017). According to GLOBOCAN 2018 estimates, a total of 1,276,106 new prostate cancer cases were reported (accounting for 7.1% of male malignant tumors), resulting in 358,989 deaths (accounting for 3.8% of all cancer fatalities in males) (Bray et al., 2018). The incidence of prostate cancer is expected to rise to 2,293,818 by 2,040 (Rawla, 2019). Although the incidence and mortality of prostate cancer are currently lower in Asia than those in Europe and the Americas, it has shown a rising trend in recent years (Chen et al., 2014; Ha Chung, Horie & Chiong, 2019), and patients with advanced cancer account for a higher proportion of new cases (Suh et al., 2021). Patients with advanced prostate cancer can receive treatments, such as androgen deprivation therapy (ADT), to achieve a certain period of clinical remission. However, most of the cases progress to castration-resistant prostate cancer (CRPC), and the curative effect for CRPC is not satisfactory (Beretta et al., 2021; Gu et al., 2021). Therefore, it is necessary to explore new treatment measures for CRPC.

The harmful effects of CRPC on patients are not only caused by the tumor itself but by distant metastases and tumor invasion to other essential organs (Schatten, 2018). It is widely recognized that in various tumors, the PI3K/AKT/mTOR signaling pathway is involved in invasion and metastasis (Hua et al., 2019; Sun & Gao, 2018). As a serine/threonine kinase, the mammalian target of rapamycin (mTOR) is considered a critical factor in tumor pathogenesis (Mossmann, Park & Hall, 2018). Studies have shown that mTOR is overexpressed in prostate cancer (Genc et al., 2019; Tan et al., 2017), and mTOR inhibitors have shown positive results in the treatment of prostate cancer in phase I clinical trials (Binal et al., 2020).

Sophocarpine, an active component extracted from the traditional Chinese herb *Sophora alopecuroides* L., has been demonstrated to possess antitumor and anti-inflammatory activities (Jiang et al., 2018). Sophocarpine was found to have the ability to inhibit the progression of hepatoma cells by downregulating the PI3K/AKT signaling pathway (Zhang et al., 2016). Furthermore, sophocarpine also possesses certain inhibitory effects on prostate cancer (Wang et al., 2017), but the underlying mechanism is unclear. Therefore, the current study aimed to verify the inhibitory effects of sophocarpine on CRPC cells and understand the underlying mechanism.

## Materials and Methods

### Reagents

Sophocarpine (HY-N0103, Purity: >98.0%) was purchased from MedChemExpress (MCE, Shanghai, China). DMEM/F12 medium was obtained from iCell Bioscience (Shanghai, China). Dulbecco’s modified Eagle’s medium (DMEM), fetal bovine serum (FBS), phosphate-buffered saline (PBS), and trypsin-EDTA were obtained from Gibco (Grand Island, NY, USA).

### Cell culture

DU145 (iCell-h250) and PC3 (iCell-h174) cell lines were purchased from iCell Bioscience (Shanghai, China). DU145 and PC3 cells were placed in 100-mm^2^ dishes, and cultured in DMEM or DMEM/F12 (1:1) complete medium (supplemented with 10% FBS and 1% penicillin/streptomycin). The culture conditions were 37 °C with 5% CO_2_. At 80–90% confluence, the cells were used for treatment or seeding. The adherent cells were collected using trypsin-EDTA.

### Cell counting kit-8 (CCK-8) assay

CCK-8 (HY-K0301; MCE, Monmouth Junction, NJ, USA) was used to evaluate cell viability, following the manufacturer’s instructions. Each well of a 96-well plate was inoculated with DU145 or PC3 cells at a density of 5 × 10^3^ cells/well. After 24 h of incubation with different concentrations of sophocarpine (0, 10, 25, 50, 75, 100, 125, 150, 175, 200, and 225 μM), 10 μL CCK-8 solution was added to each well. The absorbance of each cell group was detected at 450 nm using a microplate reader 1 h later.

### Real-time cellular analysis (RTCA)

In E-Plate 16, DU145 cells were cultured at 5 × 10^4^ cells/well, and the “cell index” parameter (which represents cell status) was automatically recorded using an RTCA System every 15 min (Zhang et al., 2019a). After adherence to the plate, the cells were treated with sophocarpine, and the “cell index” was continued to be recorded every 15 min for about 40 h.

### Colony formation assay

In 6-well plates, the two kinds of CRPC cells were plated at a density of 100–500 cells/well, and sophocarpine (0, 100, and 200 μM) was added after cell attachment. When the cells were grown to form cell colonies that were visible to the naked eye, the cells were fixed and stained using methanol and crystal violet, respectively. The cell colonies were counted and analyzed.

### Flow cytometry analysis

After 24 h of incubation with sophocarpine (100 and 200 μM), DU145 and PC3 cells were collected and resuspended. FITC Annexin V (556547; BD Biosciences, Franklin Lakes, NJ, USA) was used to detect cellular apoptotic rates following the manufacturer’s protocol.

### Wound healing assay

Scratches were made in the cell monolayer using 200 µL pipette tips after the CRPC cells (both cell lines) were grown to 90% confluence. The scratches were washed and imaged. Then, the cells were incubated in a serum-free medium with sophocarpine (0, 100, and 200 μM) for 48 h. After that, the cells were imaged again. ImageJ 1.8.0 (National Institutes of Health, Bethesda, Maryland, USA) was used to measure the scratch areas.

### Transwell invasion assay

For the Transwell invasion assay, DU145 and PC3 cells were placed into the top Transwell chambers pre-loaded with 10% Matrigel, and cultured with DMEM or DMEM/F12 media (serum-free) with sophocarpine (100 and 200 μM); DMEM or DMEM/F12 complete media (with 10% FBS) were supplied in the low chambers. About 24 h later, the invaded cells were fixed using formaldehyde (P1110; Solarbio, Beijing, China) and stained using crystal violet. The invaded cells were then counted using a microscope.

### Western blotting

The proteins from cells or animal tissues were extracted using a lysis solution (prepared using M-PER™ Mammalian Protein Extraction Reagent, Pierce Protease and Phosphatase Inhibitor Mini Tablets, 78501 and A32961, Thermo Fisher Scientific, Rockford, IL, USA). The concentrations of proteins were measured using a bicinchoninic acid (BCA) Kit (P0012; Beyotime, Jiangsu, China). The proteins were separated using 10% sodium dodecyl sulfate-polyacrylamide gel electrophoresis (SDS-PAGE) and electrotransferred to polyvinylidene difluoride (PVDF) membranes (3010040001, Sigma-Aldrich, Mannheim, Germany), which were subsequently blocked with skimmed milk (1172GR500; BioFroxx). Then, the membranes were incubated with the following specific antibodies overnight: GAPDH (10494-1-AP, 1:5,000; Proteintech, Rosemont, IL, USA), Ki67 (ab16667, 1:1,000; Abcam, Cambridge, UK), Bcl-2 (12789-1-AP, 1:1,000; Proteintech, Rosemont, IL, USA), Bax (50599-2-Ig, 1:1,000; Proteintech, Rosemont, IL, USA), α-SMA (AF1032, 1:1,000, Affinity), N-cadherin (ab76011, 1:1,000; Abcam, Cambridge, UK), collagen I (AF7001, 1:1,000; Affinity), p-AKT (4060, 1:2,000; CST), AKT (4691, 1:1,000; CST), p-mTOR (ab109268, 1:1,000; Abcam, Cambridge, UK), mTOR (20657-1-AP, 1:1,000; Proteintech, Rosemont, IL, USA), and PI3K (A0982, 1:1,000; ABclonal). After 1 h of incubation with a secondary goat anti-rabbit/mouse antibody (SA00001-2 and SA00001-1, 1:5,000; Proteintech, Rosemont, IL, USA), the membranes were exposed to the enhanced chemiluminescence (ECL) reagent (Thermo Scientific, Waltham, MA, USA) and visualized using an autoradiographic film. The protein concentrations were measured using ImageJ 1.8.0 and normalized to that of GAPDH (p-AKT and p-mTOR expression were normalized to that of AKT or mTOR, respectively).

### Immunofluorescence analysis

CRPC cells were placed on coverslips in 6-well plates and treated with sophocarpine (0 and 200 μM) after adhering. Subsequently, the cells were fixed using 4% paraformaldehyde and permeabilized with 0.5% Triton X-100 (T8200; Solarbio, Beijing, China) and blocked with 10% goat serum (AR0009; Boster, Hubei, China). The coverslips were incubated with specific antibodies: Ki67 (ab16667, 1:100; Abcam, Cambridge, UK), N-cadherin (22018-1-AP; 1:100, Proteintech, Rosemont, IL, USA), collagen I (AF7001, 1:100; Affinity), and p-mTOR (67778-1-Ig, 1:100; Proteintech, Rosemont, IL, USA). After washing thrice, the cells were incubated with a fluorescence-conjugated secondary antibody (SA00013-1 and SA00013-2, 1:400; Proteintech, Rosemont, IL, USA) at 37 °C for 1 h. Mounting Medium with DAPI (S2110; Solarbio, Beijing, China) was used to mount the coverslips on the glass slides. A fluorescence microscope was used to image the coverslips.

### Animal experiments

Ten male BALB/c nude mice were obtained from the Laboratory Animal Center of Wenzhou Medical University (Zhejiang, China). The experimental protocol was approved by the Laboratory Animal Ethics Committee of Wenzhou Medical University (Number: wydw2021-0200). The mice were reared in cages at 25 °C and 50% humidity with a 12 h light/dark cycle, and provided with sufficient food and water. The animals were acclimatized for at least 1 week before the experiments. During the experiment, the survival status of mice was checked twice a week.

DU145 cells (5 × 10^6^ cells) in 100 μL PBS were injected into the subcutaneous space of the inner thigh of the nude mice. The nude mice that could not form tumors were not used in this experimental study. After the formation of xenograft tumors, the mice were randomly assigned to two groups (N = 5 per group) according to the random number method. The sophocarpine-treated group received intraperitoneal injections of sophocarpine (35 mg/kg, dissolved in PBS) twice a week, and the other group (the negative control group) received PBS of the same dose. All the mice were kept under the same conditions throughout the experiment. After 4 weeks, all the mice were alive and euthanized using an intraperitoneal injection of sodium pentobarbital (50 mg/kg). The tumors were resected and imaged. The volumes and weights of the tumors were measured, and the tumor proteins were measured using western blotting, as described above.

### Statistics analysis

The results are presented as mean ± SD. A *P*-value < 0.05 indicated statistical significance. The groups were compared using one-way ANOVA (three groups) or t-test (two groups) using GraphPad Prism 9 (GraphPad Software Inc, San Diego, CA, USA).

## Results

### Sophocarpine inhibited the proliferation of CRPC cells

**Figure 1:**
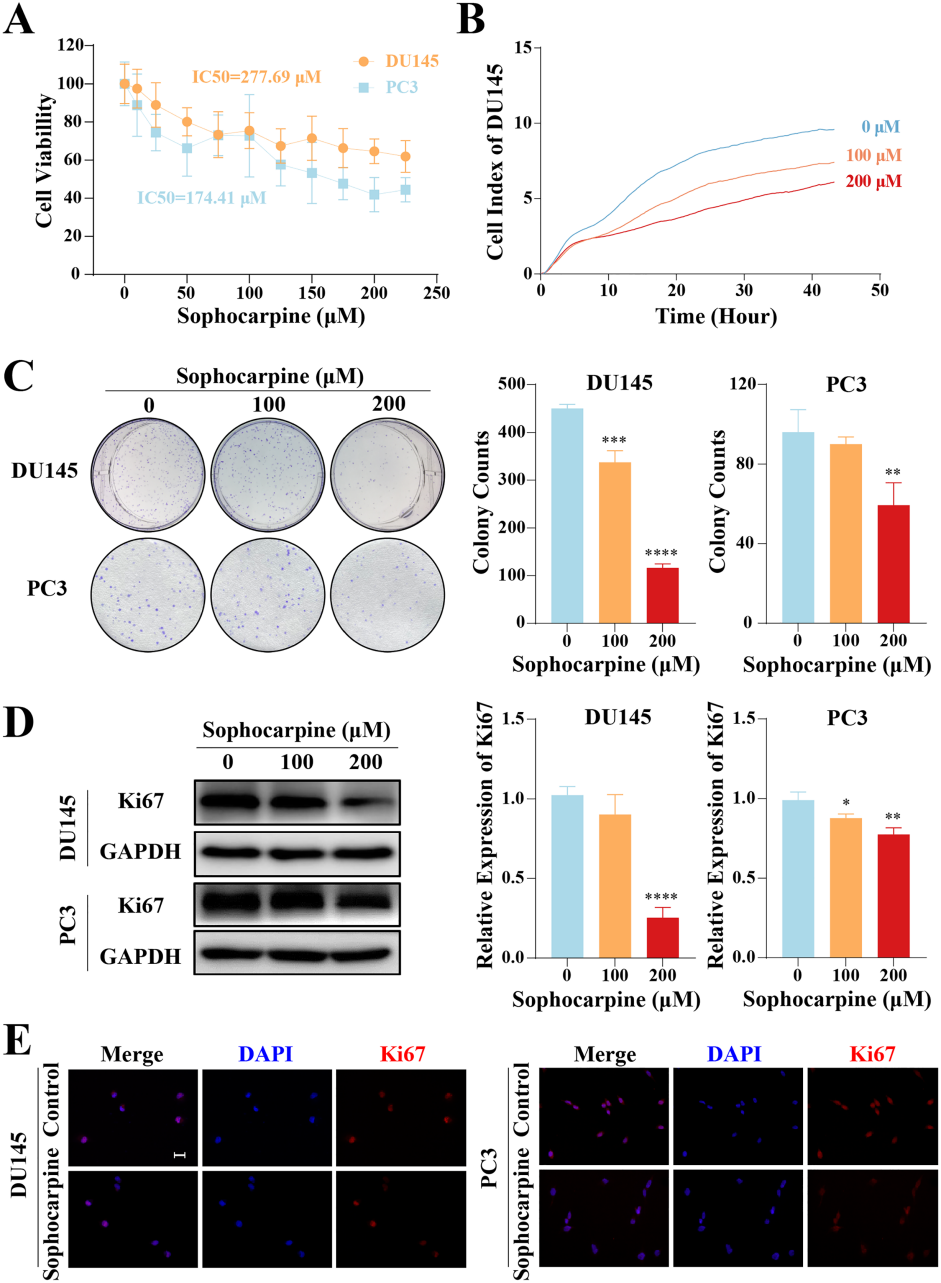
Sophocarpine inhibited the proliferation of CRPC cells. (A) After treatment with different concentrations of sophocarpine (0, 10, 25, 50, 75, 100, 125, 150, 175, 200, and 225 μM), the viability of DU145 and PC3 cells were analyzed using CCK-8 analysis. (B) Growth curves show the cell proliferation ability of DU145 after sophocarpine administration. Cell index was determined using RTCA. (C) For the colony formation assay, colonies were counted to evaluate the inhibitory effect of sophocarpine on the growth of DU145 and PC3 cells. (D) Ki67 expression measured by western blotting reflected the proliferative status of DU145 and PC3 cells. (E) The immunofluorescence staining of Ki67 in DU145 and PC3 cells showed changes in Ki67 expression after sophocarpine administration (0 and 200 μM). Scale bar = 20 μm. *P < 0.05; **P < 0.01; ***P < 0.001; ****P < 0.0001.

The inhibitory effect of sophocarpine on CRPC cells was preliminarily evaluated using the CCK-8 assay. The cell viability of both types of cells showed a sophocarpine-dose-dependent decrease (Fig. 1A). Based on the IC50 values (DU145 IC50 = 277.69 μM, PC3 IC50 = 174.41 μM) of the two cell lines, we chose 100 and 200 μM concentrations of sophocarpine for the following experiments.

DU145 cell growth was evaluated using RTCA. The cell indices of the treatment groups (100 and 200 μM sophocarpine) decreased significantly (Fig. 1B). The colony formation assay results showed that the DU145 cells grew fewer colonies after the administration of sophocarpine (450.00 ± 8.89 colonies for the control group, 337.00 ± 24.33 colonies for the 100 μM sophocarpine group, and 116.33 ± 8.33 colonies for the 200 μM sophocarpine group). The colonies of the PC3 cells reduced in the 200 μM sophocarpine group (59.33 ± 11.24 colonies) compared to the control group (96.00 ± 11.36 colonies). The 100 μM sophocarpine group (90 ± 3.61 colonies) showed no significant difference compare to the control group (Fig. 1C).

Ki67 is a type of antigen associated with proliferating cells, the expression level of which indicates the proliferative activity of the cells (Guo et al., 2021; Zhang et al., 2019b). The western blotting results showed that the expression of Ki67 reduced after treatment with sophocarpine in both DU145 and PC3 cells (Fig. 1D). The immunofluorescence assay showed similar results (Fig. 1E). These findings indicated that the proliferation of CRPC cells was suppressed by sophocarpine.

### Sophocarpine induced apoptosis in CRPC cells

**Figure 2:**
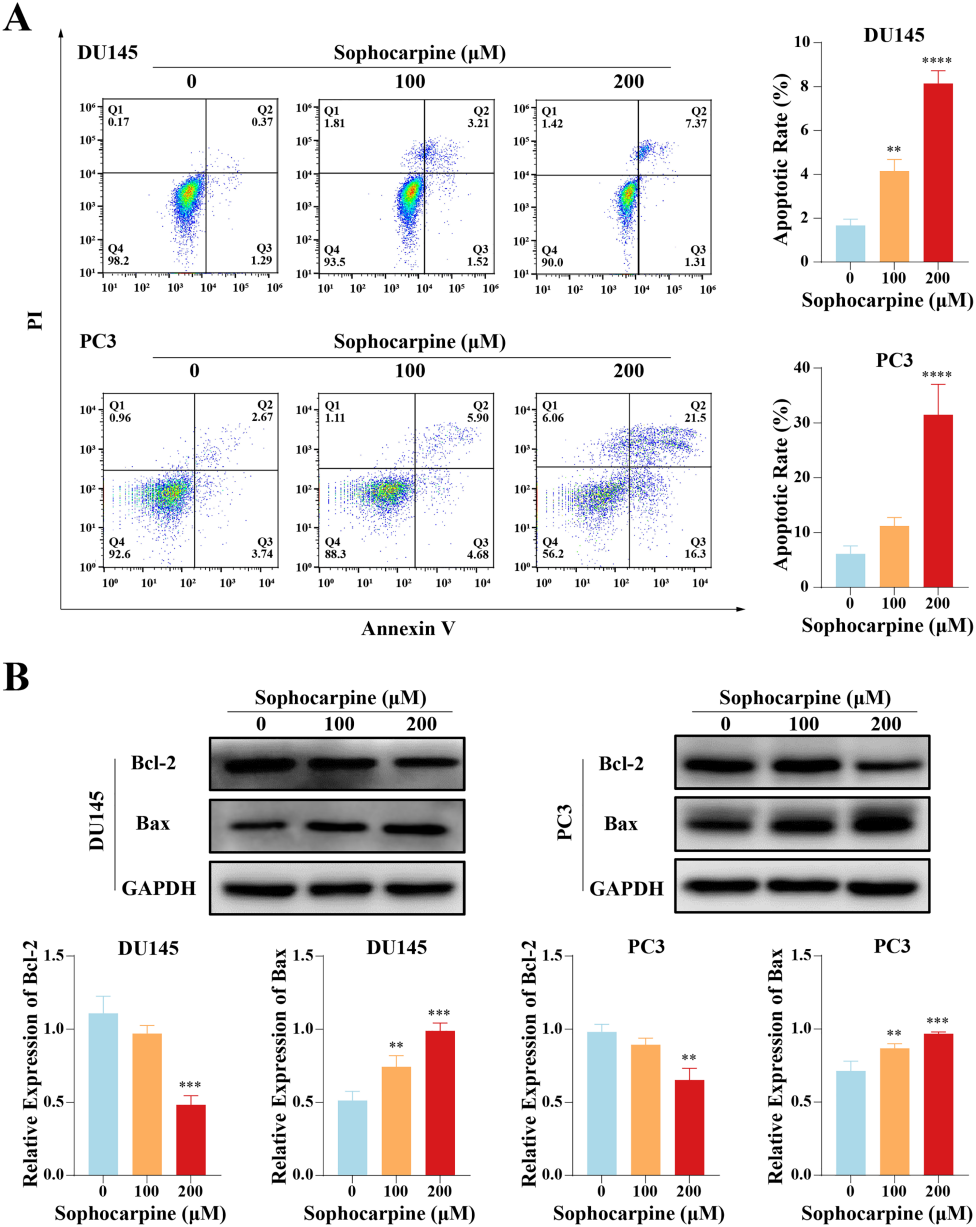
Sophocarpine promoted apoptosis of CRPC cells. (A) In sophocarpine-treated DU145 and PC3 cells, the apoptotic cells were counted using flow cytometry analysis, to measure the apoptosis-inducing ability of sophocarpine. (B) Western blotting was performed to detect the changes in Bcl-2 and Bax expression in DU145 and PC3 cells after sophocarpine administration. **P < 0.01; ***P < 0.001; ****P < 0.0001.

Flow cytometry was performed to evaluate cell apoptotic rates. After treatment with different concentrations of sophocarpine, DU145 cells showed dose-dependent apoptosis (1.67 ± 0.29% for the control group, 4.15 ± 0.53% for the 100 μM group, and 8.14 ± 0.58% for the 200 μM group). In contrast, in PC3 cells, only the higher sophocarpine concentration group showed a significant increase in the apoptosis rate (6.11 ± 1.45% for the control group, 11.19 ± 1.57% for the 100 μM group, and 31.45 ± 5.58% for the 200 μM group) (Fig. 2A). We also measured the levels of apoptosis-related proteins Bcl-2 and Bax using western blotting (Fernandes et al., 2021; Garner et al., 2016). We found that sophocarpine decreased the level of Bcl-2 and increased that of Bax (Fig. 2B), indicating that sophocarpine induced apoptosis in both CRPC cell lines through a mitochondrial-dependent pathway.

### Sophocarpine reduced migration and invasion of CRPC cells

**Figure 3:**
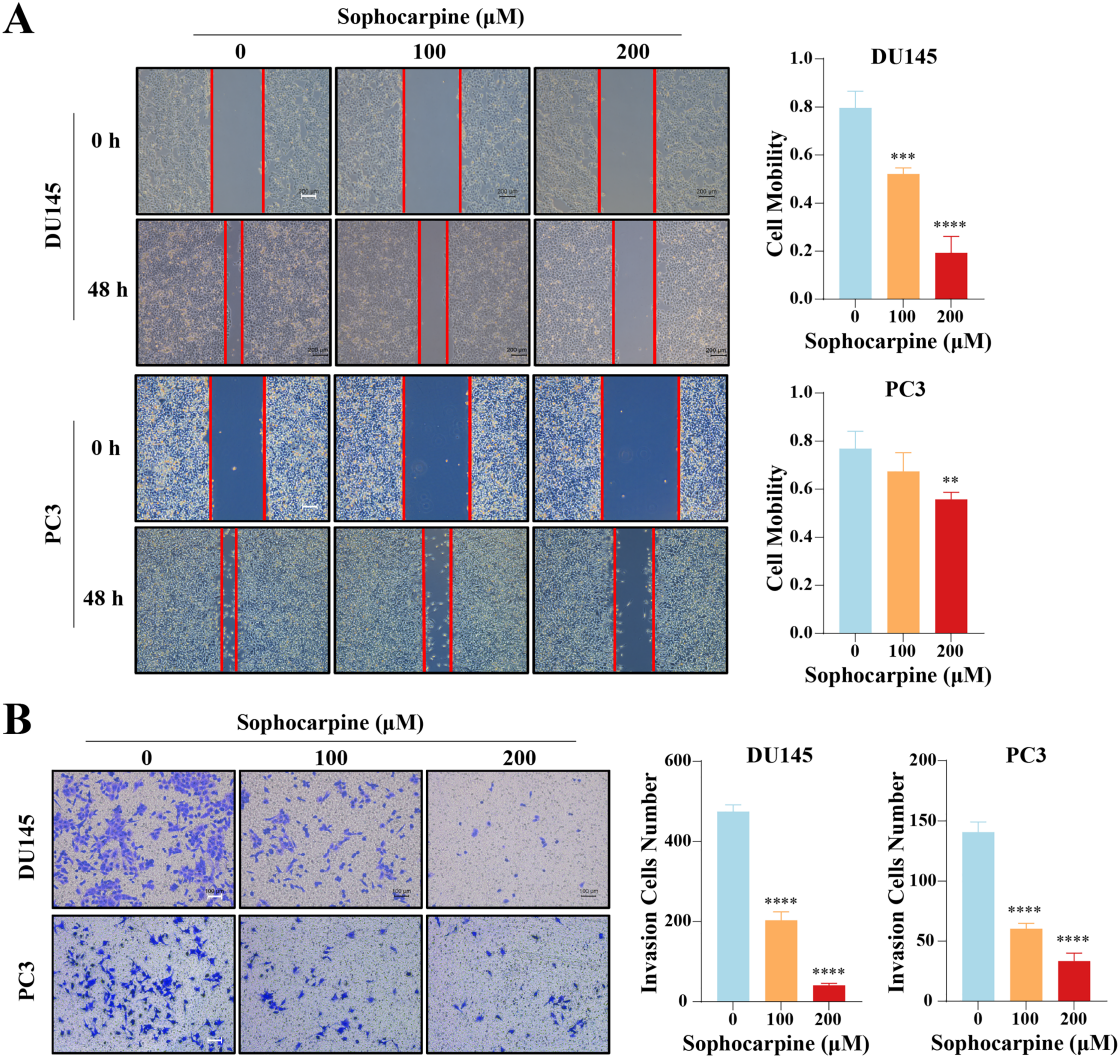
Sophocarpine suppressed the migration and invasion abilities of CRPC cells. (A) For the wound healing assay, scratches were made in the monolayer of DU145 and PC3 cells. The cells were incubated with different concentrations of sophocarpine (0, 100 and 200 μM) for 48 h (scale bar = 200 μm). Cell mobility = (scratch area at 0 h) − (scratch area at 48 h)/(scratch area at 0 h). (B) The invasion in DU145 and PC3 cells were evaluated using the Transwell invasion assay. The cells were incubated with sophocarpine for 24 h. The numbers of invaded cells reflected the invasion ability of the cells. Scale bar = 100 μm. **P < 0.01; ***P < 0.001; ****P < 0.0001.

The effect of sophocarpine on cell migration and invasion was evaluated using wound healing and Transwell invasion assays, respectively. In the DU145 cells, the 200 μM sophocarpine group (19.33 ± 6.82%) and the 100 μM sophocarpine group (52.15 ± 2.49%) showed smaller healing areas compared to the control group (79.65 ± 6.90%). PC3 cells in the 200 μM group (55.69 ± 3.01%) also showed smaller migration areas, while the 100 μM group (67.36 ± 7.80%) showed no significant difference compared to the control group (76.90 ± 7.17%) (Fig. 3A). The Transwell invasion assay was conducted to determine the invasion abilities of the cells. The number of invaded cells decreased significantly after treatment with sophocarpine (474.25 ± 17.11 cells for the control group, 203.00 ± 21.18 cells for the 100 μM group, and 40.25 ± 5.32 cells for the 200 μM group of DU145 cells; 140.70 ± 8.51 cells for the control group, 60.33 ± 4.51 cells for the 100 μM group, and 33.33 ± 6.66 cells for the 200 μM group of PC3 cells) (Fig. 3B).

### Sophocarpine suppressed the epithelial-mesenchymal transition (EMT) process in CRPC cells

**Figure 4:**
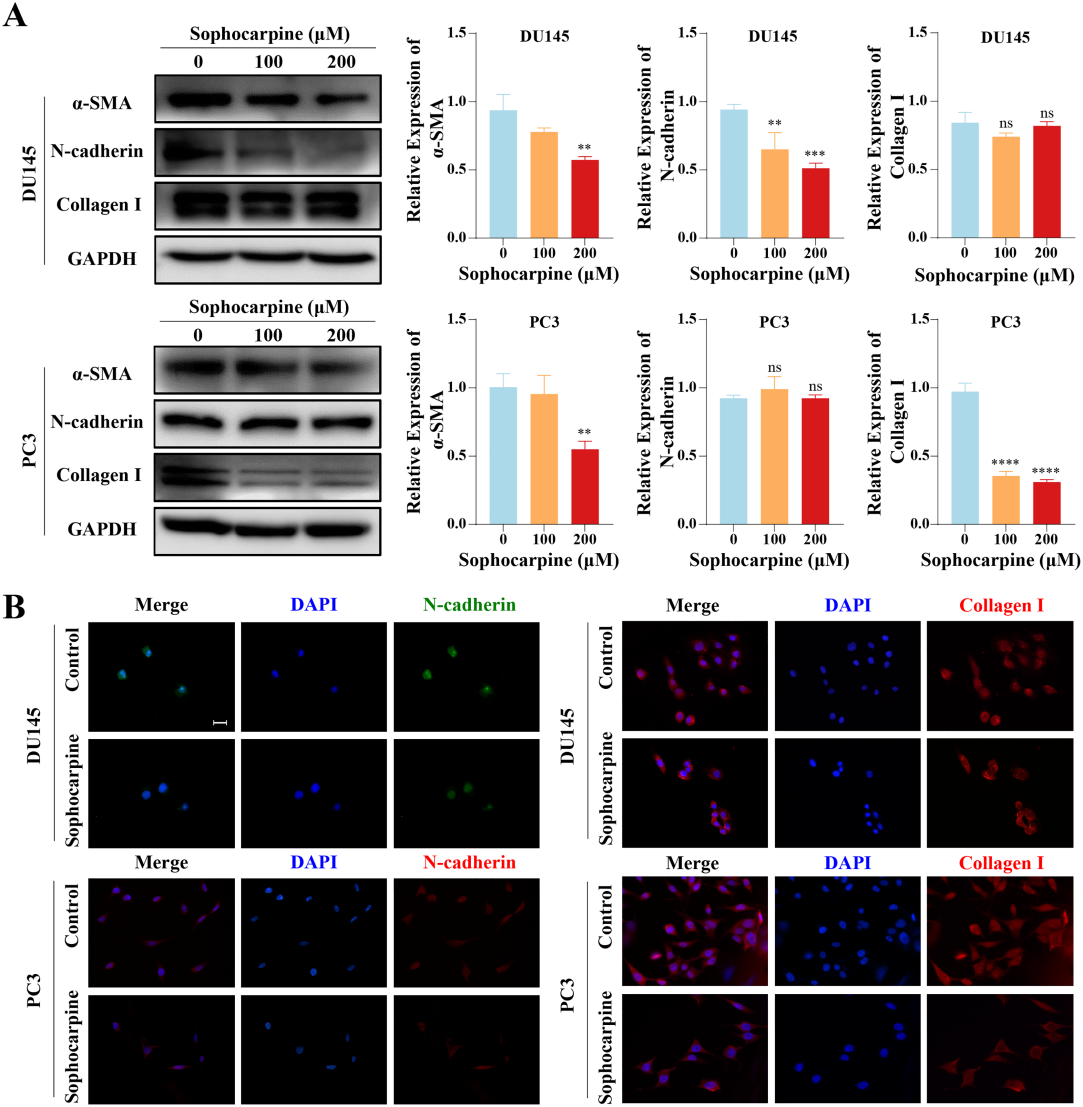
Sophocarpine inhibited EMT progression in CRPC cells. (A) EMT-related protein (α-SMA, N-cadherin and collagen I) expression in DU145 and PC3 cells measured using western blotting, followed by the grayscale analysis charts. (B) The immunofluorescence staining of N-cadherin and collagen I in DU145 and PC3 cells after treatment with sophocarpine (0 and 200 μM). Scale bar = 20 μm. **P < 0.01; ***P < 0.001; ****P < 0.0001; “ns” represents not significant.

We measured the levels of EMT-related proteins. Sophocarpine reduced the level of α-SMA in both cell lines. In DU145 cells, sophocarpine reduced N-cadherin expression, though no significant difference was observed in collagen I expression. However, in PC3 cells, the level of collagen I reduced after treatment with sophocarpine, and there was no change in N-cadherin expression (Fig. 4A). The immunofluorescence assay showed similar results (Fig. 4B). These data revealed that sophocarpine regulated EMT and inhibited cell invasion and migration.

### Sophocarpine inactivated the PI3K/AKT/mTOR signaling pathway in CRPC cells

**Figure 5:**
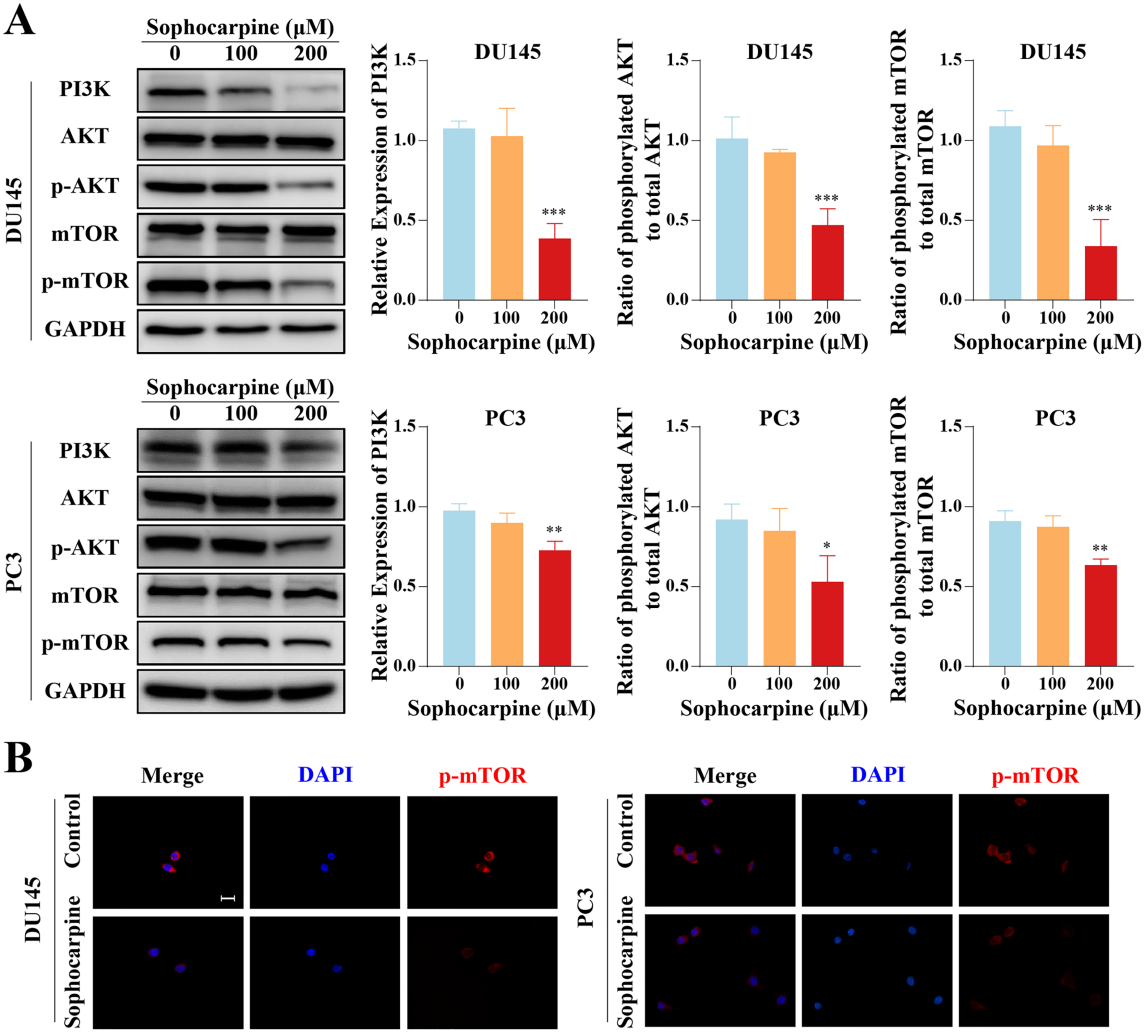
The inhibitory effect of sophocarpine on the PI3K/AKT/mTOR signaling pathway proteins in CRPC cells. (A) The expression of PI3K and the phosphorylation of AKT and mTOR in DU145 and PC3 cells were measured using western blotting, followed by the grayscale analysis charts (p-AKT and p-mTOR expression were normalized to that of AKT and mTOR, respectively). (B) The immunofluorescence staining of p-mTOR in DU145 and PC3 cells after treatment with sophocarpine (200 μM). Scale bar = 20 μm. *P < 0.05; **P < 0.01; ***P < 0.001.

Western blotting was used to measure the levels of proteins of the PI3K/AKT/mTOR signaling pathway in CRPC cells. Sophocarpine, at a higher concentration (200 μM), decreased PI3K expression. As for the downstream proteins, AKT and mTOR were found to be expressed at low phosphorylated levels (Fig. 5A). The expression of phosphorylated mTOR was measured using immunofluorescence. mTOR was expressed in the cytoplasm and inhibited by sophocarpine (Fig. 5B).

### *In vivo* tumor progression was suppressed after sophocarpine treatment

**Figure 6:**
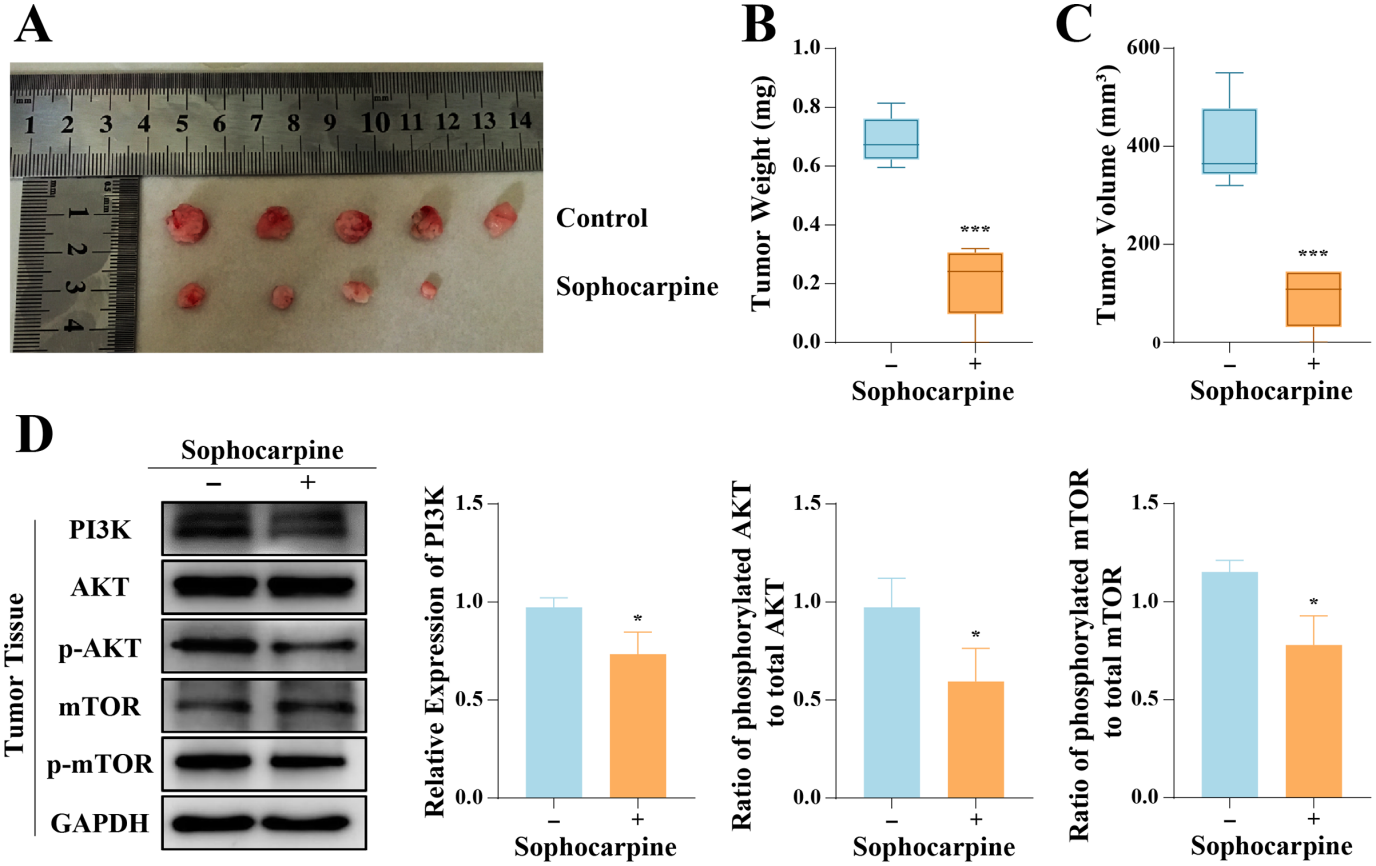
Sophocarpine suppressed the progression of xenograft tumors *in vivo*. (A) The appearance of xenograft tumors in nude mice injected with DU145 cells. (B, C) The weights and volumes of tumors were measured to detect the effect of sophocarpine *in vivo*. (D) The expression of PI3K and the phosphorylation of AKT and mTOR in tumors were determined using western blotting, followed by the grayscale analysis charts (p-AKT and p-mTOR expression were normalized to that of AKT or mTOR, respectively). *P < 0.05; ***P < 0.001.

The inhibitory effect of sophocarpine on DU145 cells was verified *in vivo*. All 10 nude mice inoculated with DU145 cells formed tumors, and 4-week administration of sophocarpine reduced tumor growth (Fig. 6A), decreased the tumor weights (0.21 ± 0.13 g, compared to 0.69 ± 0.08 in the control group) (Fig. 6B), and reduced the tumor volumes (91.70 ± 61.23 mm^3^, compared to 400.80 ± 88.66 mm^3^ in the control group) (Fig. 6C). After with the administration of sophocarpine, the tumor in one nude mouse was invisible to the naked eye, and its weight and volume were recorded as 0 mg and 0 mm^3^, respectively. Western blotting was performed to measure the PI3K/AKT/mTOR pathway protein expression in tumor tissues. The results were consistent with those of the cellular protein analysis (Fig. 6D).

## Discussion

Almost all patients with prostate cancer progress to CRPC after ADT (Wang et al., 2018). Current treatments for CRPC, including taxane-based chemotherapy and anti-androgen drugs, provide limited survival benefits (Ma et al., 2021; Ojo et al., 2015). Attention has shifted to the application of natural medicine as research has overcome the technical barriers to the isolation of natural compounds (Atanasov et al., 2021; Newman & Cragg, 2020). In traditional Chinese medicine, *Sophora alopecuroides* L. is widely used due to its various effects, such as dispelling rheumatism and detoxification (Wang et al., 2013). In the current study, we found that sophocarpine, a bioactive component extracted from *Sophora alopecuroides* L, had an inhibitory effect on the progression of CRPC cells. *In vitro*, sophocarpine administration inhibited CRPC cell proliferation, induced apoptosis, and impeded cell invasion and migration. *In vivo*, sophocarpine inhibited the growth of xenograft tumors. Although there are few reports on the clinical application of sophocarpine, the antitumor effect of the alkaloid has been continuously explored (Li et al., 2020). Wang et al. (2019) found that sophocarpine inhibited the migration of colorectal cancer cells by downregulating the MEK/ERK/VEGF pathway. Huang et al. (2019) found that in gastric cancer cells, sophocarpine caused autophagy by arresting the cell cycle at the G0/G1 phase, and induced apoptosis by inhibiting the PI3K/AKT signaling pathway. In addition, Zhang et al. (2008) reported the anti-cachexia effect of sophocarpine by inhibiting TNF-α and IL-6.

EMT is a key process in tumor progression. During EMT, epithelial tumor cells lose the epithelial cell hallmarks and acquire mesenchymal characteristics (Zeisberg & Neilson, 2009), such as increased expressions of extracellular matrix (ECM) proteins and migratory properties (Sisto, Ribatti & Lisi, 2021). N-cadherin (a cell adhesion molecule) and α-SMA (one of the cytoskeletal proteins) are mesenchymal marker proteins, which are considered important mediators in the EMT process and tumor cell migration (Huang et al., 2021; Janthamala et al., 2021). In the current study, we found that α-SMA and N-cadherin were downregulated by sophocarpine in DU145 cells, and α-SMA and collagen I (one of the ECM proteins) were downregulated in the PC3 cells. The decreased expression of the mesenchymal markers and ECM proteins indicated that sophocarpine suppressed the transformation of these cells into mesenchymal cells, suggesting that it inhibited the EMT process. Similar phenomena were also observed in other tumor types. Liu et al. (2017) found that in head and neck cancer, sophocarpine upregulated E-cadherin expression (epithelial marker) and decreased vimentin expression (mesenchymal marker). Yang, Wen & Zhao (2021) demonstrated that in colon cancer cells, sophocarpine reversed the EMT process by upregulating the expression of E-cadherin and downregulating the expression of N-cadherin and vimentin. All these results suggested that sophocarpine might inhibit cell migration and invasion by inhibiting EMT.

In multiple cancers including prostate cancer, the PI3K/AKT/mTOR signaling pathway was found to be aberrantly activated (Pungsrinont, Kallenbach & Baniahmad, 2021). The aberrant activation was also found in CRPC and was shown to play an essential role in tumor progression (Yu et al., 2021). When the PI3K/AKT/mTOR signaling pathway is activated, mTOR is phosphorylated and acts as a protein kinase, affecting the synthesis of proteins and regulating cellular growth, metabolism, and migration by phosphorylating several downstream signaling proteins, such as S6K1, 4E-BP1 and PKC (Hua et al., 2019). The PI3K/AKT/mTOR signaling pathway also impacts the EMT process. EMT causes the remodeling of the cytoskeleton and formation of lamellipodia, which can be suppressed by the inhibition of mTOR, thereby weakening the abilities of migration and invasion (Karimi Roshan et al., 2019). In addition, cellular apoptosis is also regulated by the PI3K/AKT/mTOR pathway. Mohan et al. (2016) found that the apoptosis of tumor cells could be induced by targeting the PI3K/AKT/mTOR pathway. In the current study, the suppression of the PI3K/AKT/mTOR signaling pathway by sophocarpine was observed in CRPC cells. Sophocarpine downregulated PI3K expression and reduced the level of p-AKT and p-mTOR. These results suggested an essential role of the PI3K/AKT/mTOR pathway in the antitumor effects of sophocarpine against CRPC. Huang et al. (2019) reported that sophocarpine decreased PI3K and p-AKT protein expression in gastric cancer cells, and Jiang et al. (2018) showed that sophocarpine decreased PI3K expression and AKT phosphorylation in the hepatic tissue of LPS-induced mice, suggesting that sophocarpine exerted its therapeutic effect by regulating the PI3K/AKT pathway.

## Conclusions

**Figure 7:**
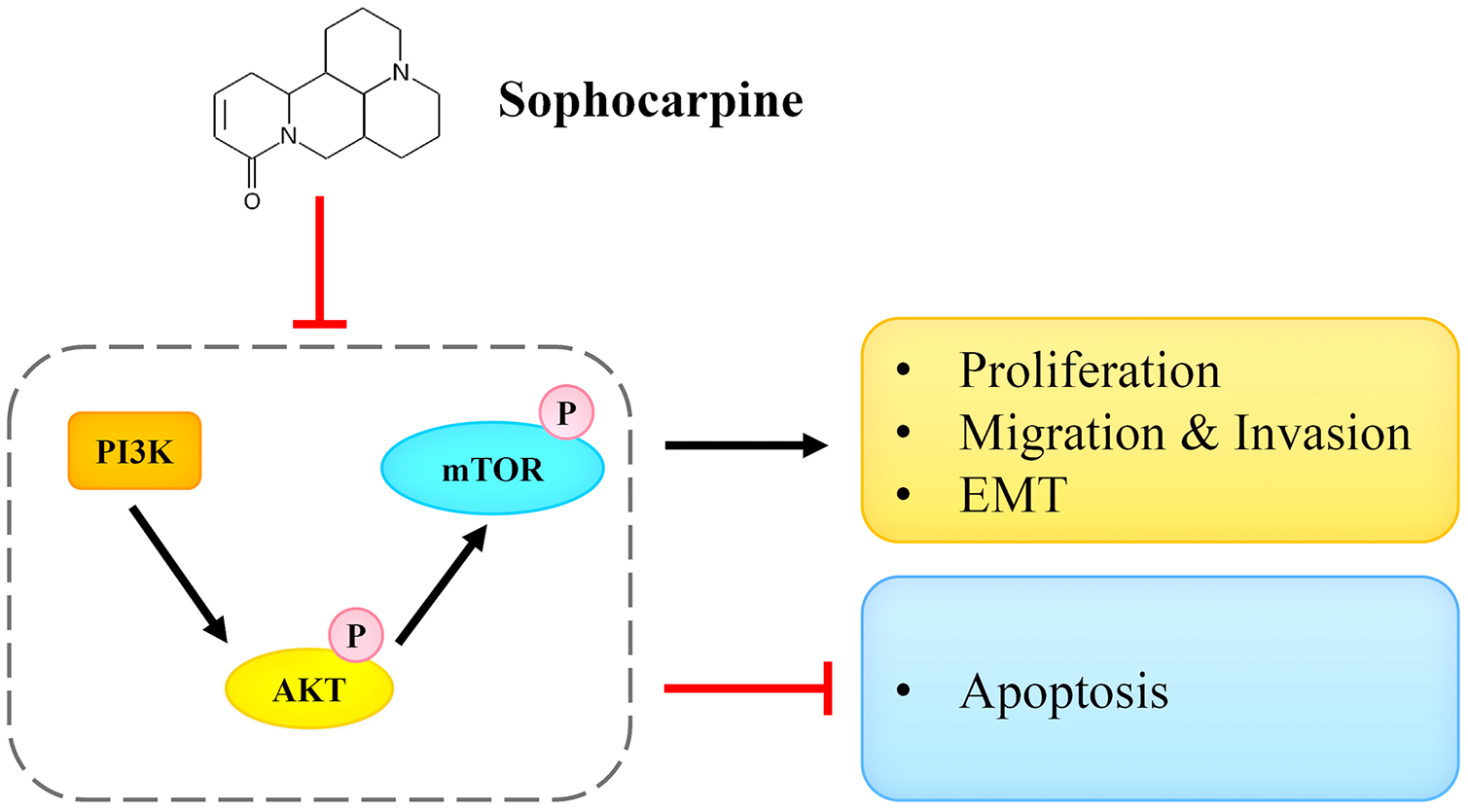
Sophocarpine exerted its inhibitory effect on CRPC cells by antagonizing the PI3K/AKT/mTOR signaling pathway. Sophocarpine suppressed the PI3K/AKT/mTOR pathway to exert its antitumor effect. Black line, promotion; red line, inhibition.

In conclusion, we reported that sophocarpine suppressed cell proliferation, induced cell apoptosis, and inhibited migration and invasion of CRPC cells. The potential underlying mechanism may be associated with the targeting and the inactivation of the PI3K/AKT/mTOR signaling pathway (Fig. 7). This finding may provide novel ideas for CRPC treatment.

## Supplemental Information

10.7717/peerj.14042/supp-1 Supplemental Information 1 Raw data of quantitative data. (Sheet 1) CCK-8. (Sheet 2) RTCA. (Sheet 3) Colonies numbers. (Sheet 4) Cell apoptotic rate. (Sheet 5) Wound healing assay and Transwell invasion assay. (Sheet 6) Tumor volume and weight. (Sheet 7) Western Blotting.

10.7717/peerj.14042/supp-2 Supplemental Information 2 Raw data of image files. The images of colony formation assay, immunofluorescence analysis, flow cytometry, wound healing assay, Transwell invasion assay, western blotting, and molecular docking analysis.

10.7717/peerj.14042/supp-3 Supplemental Information 3 Author Checklist.

## Additional Information and Declarations

### Data Availability

The following information was supplied regarding data availability: The raw data is available in the Supplemental Files.
