# Supplementary material for: Sophocarpine inhibits tumor progression by antagonizing the PI3K/AKT/mTOR signaling pathway in castration-resistant prostate cancer
Source: PeerJ. 2022 Sep 16;10:e14042. doi: 10.7717/peerj.14042 (PMC9484452; doi:10.7717/peerj.14042)
Supplement: Supplemental Information 2 — The images of colony formation assay, immunofluorescence analysis, flow cytometry, wound healing assay, Transwell invasion assay, western blotting, and molecular docking analysis. [file peerj-10-14042-s002.docx]

**Raw Data**

**Figure 1**

**（C）Colony Formation Assay**

**DU145**
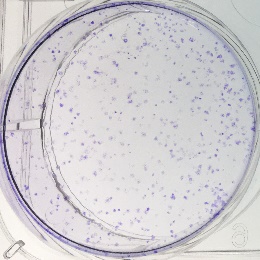

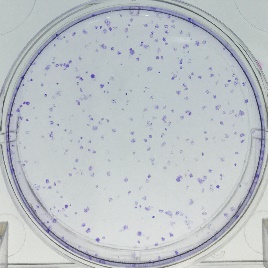

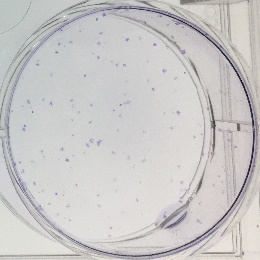


**PC3**
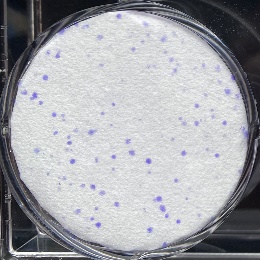

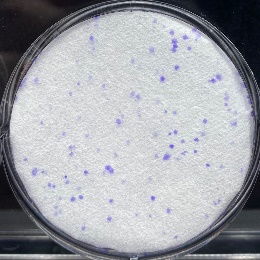

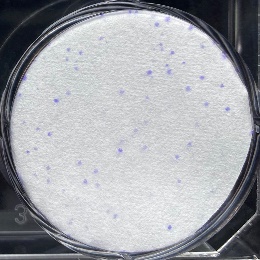


**（D）Western Blotting**

**DU145**

Ki67
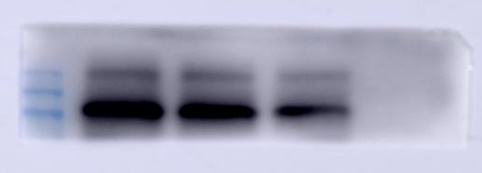


GAPDH
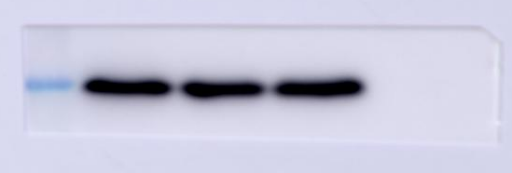


**PC3**

Ki67
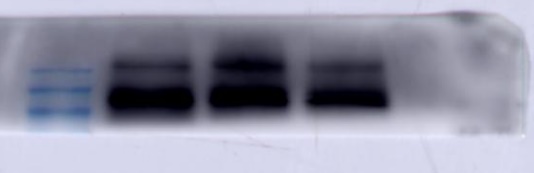


GAPDH
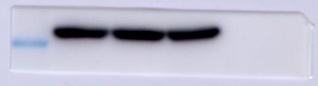


**(E) Immunofluorescence Analysis**

**DU145 (Ki67)**

Control
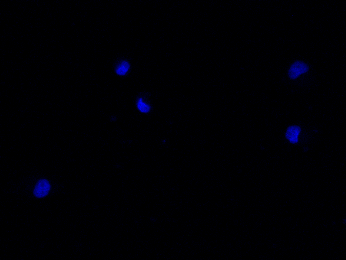

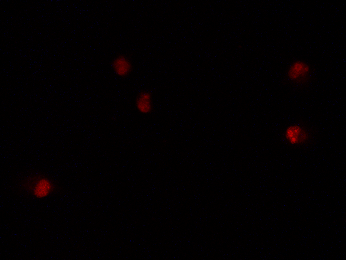


Sophocarpine
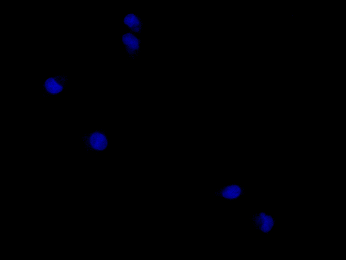

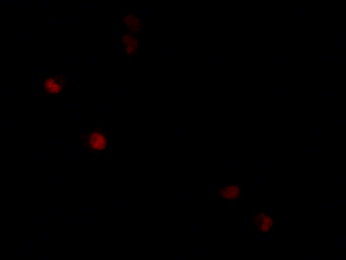


**PC3 (Ki67)**

Control
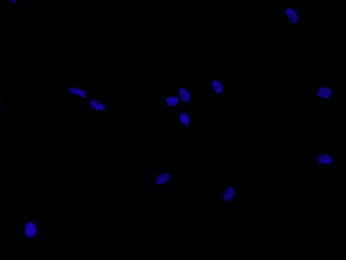

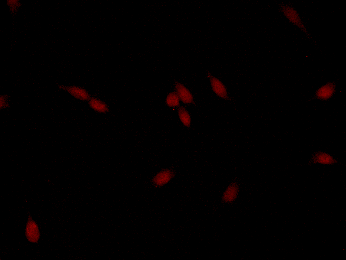


Sophocarpine
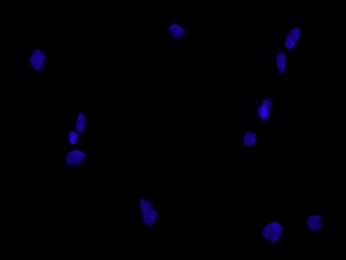

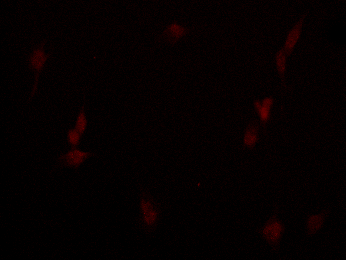


**Figure 2**

**(A) Flow Cytometry Analysis**

**DU145**
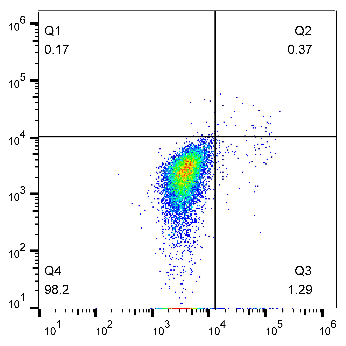

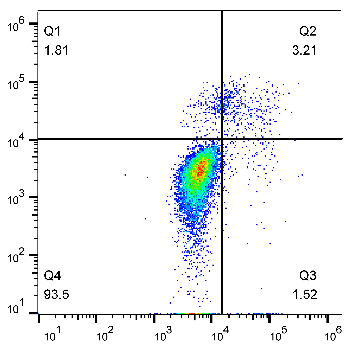

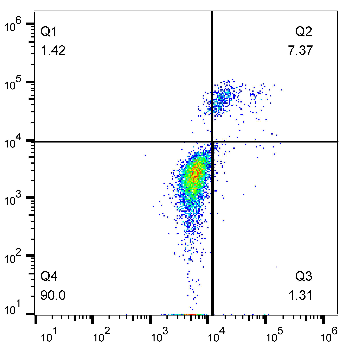


**PC3
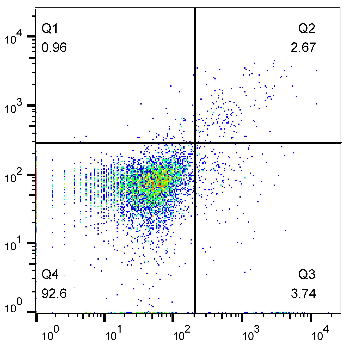

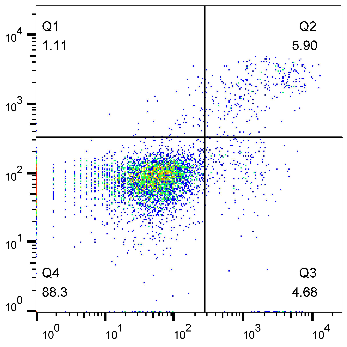

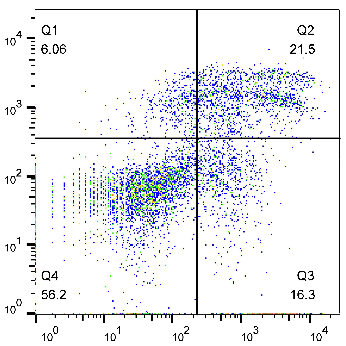
**

**（B）Western Blotting**

**DU145**

Bcl-2
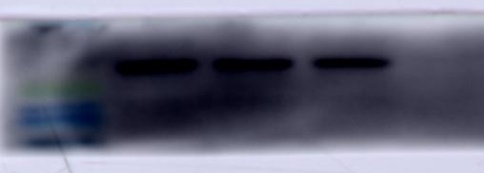


Bax
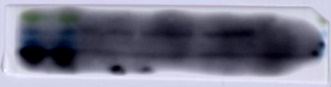


GAPDH
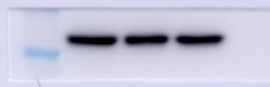


**PC3**

Bcl-2
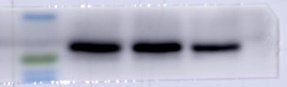


Bax
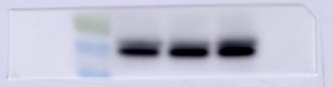


GAPDH
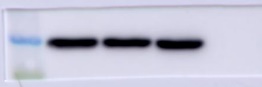


**Figure 3**

**（A）Wound Healing Assay**

**DU145**

0 h
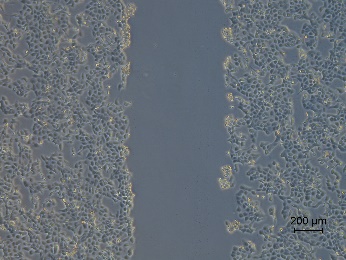

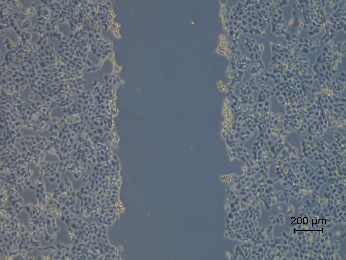

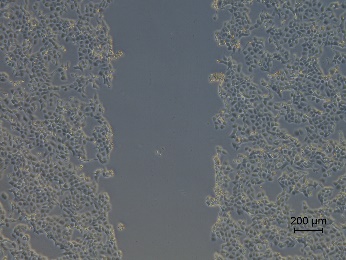


48h
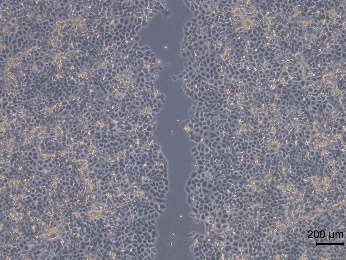

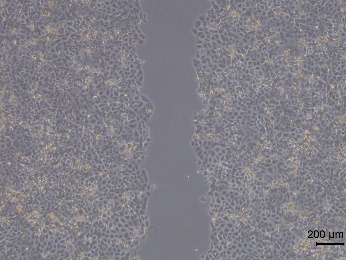

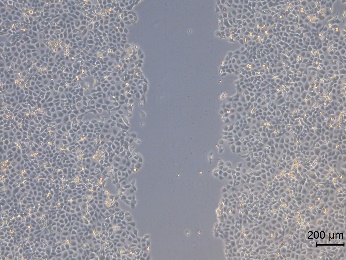


**PC3**

0 h
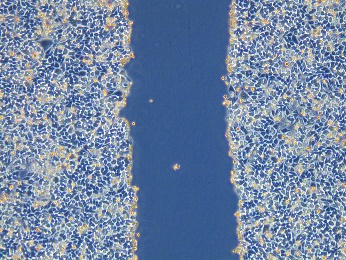

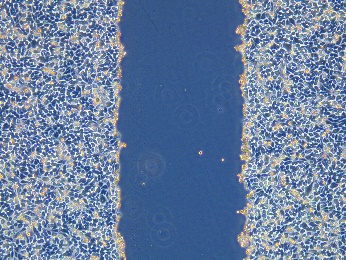

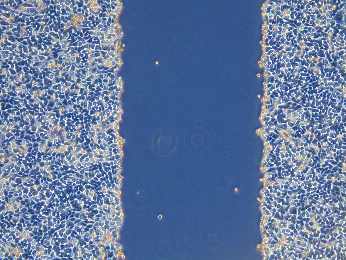


48h
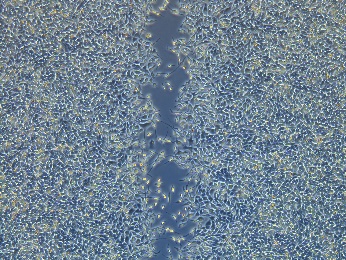

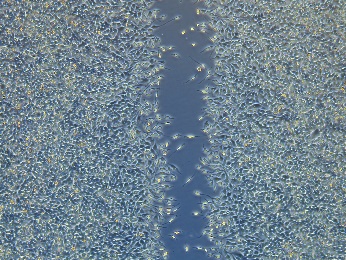

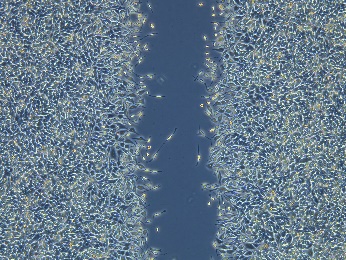


**（B）Transwell Invasion Assay**

**DU145
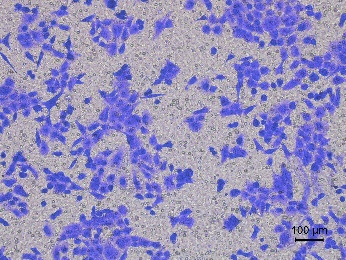

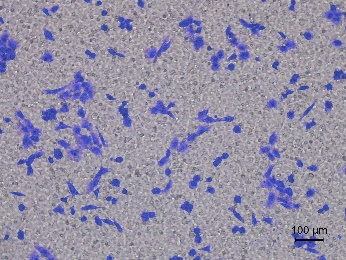

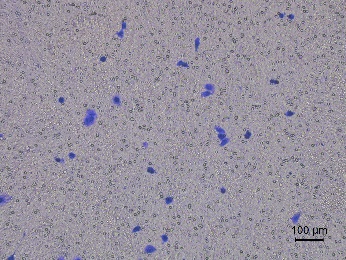
**

**PC3
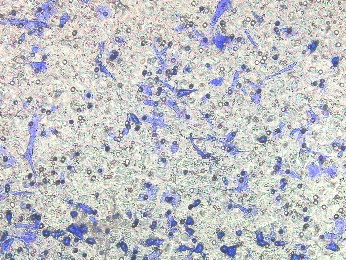

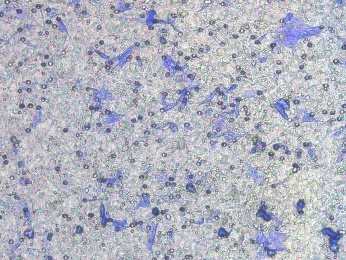

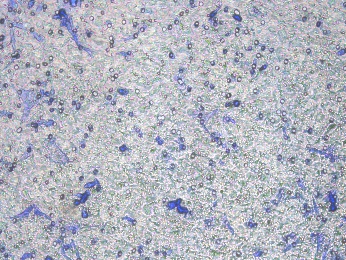
**

**Figure 4**

**（A）Western Blotting**

**DU145**

α-SMA
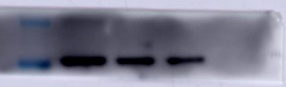


N-cadherin
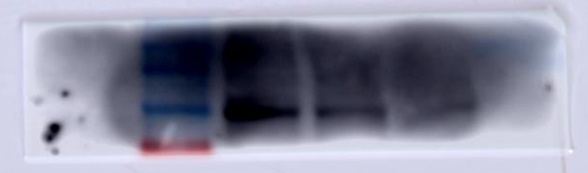


Collagen I
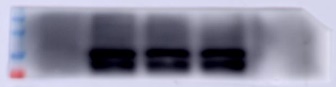


GAPDH
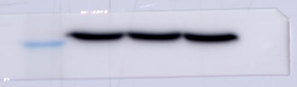


**PC3**

α-SMA
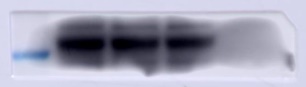


N-cadherin
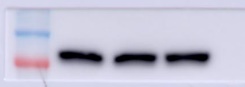


Collagen I
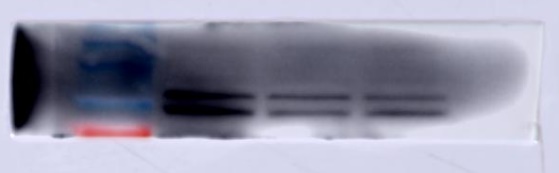


GAPDH
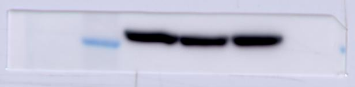


**(B) Immunofluorescence Analysis**

**DU145 (N-cadherin)**

Control
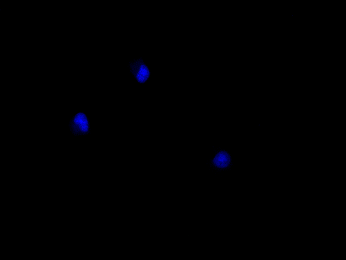

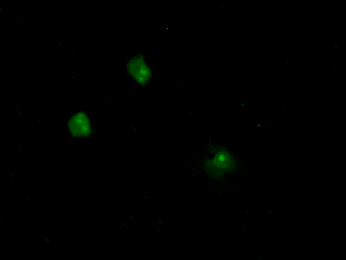


Sophocarpine
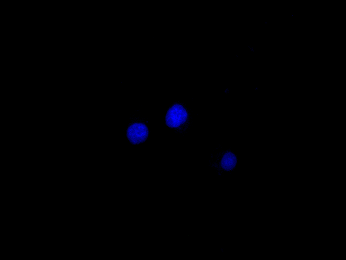

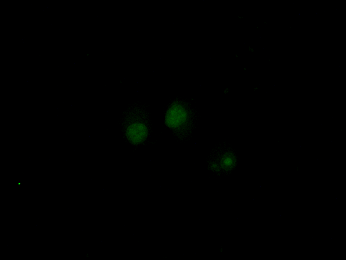


**PC3 (N-cadherin)**

Control
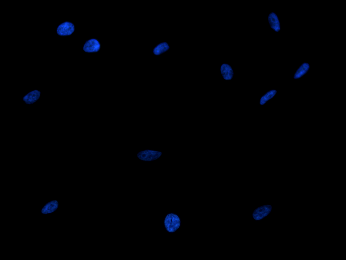

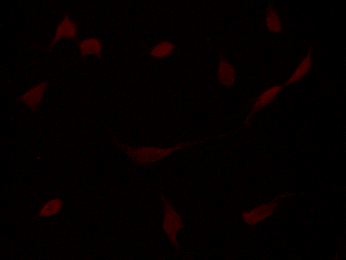


Sophocarpine
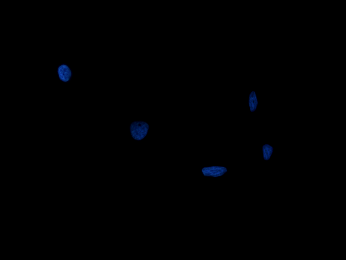

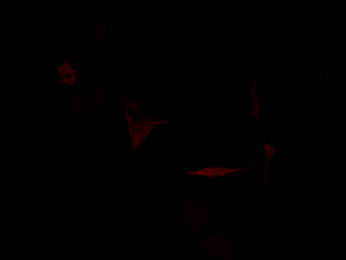


**DU145 (Collagen I)**

Control
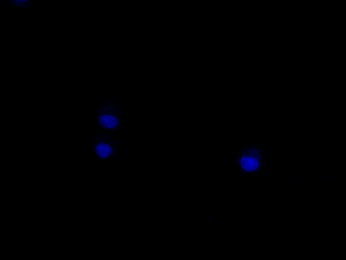

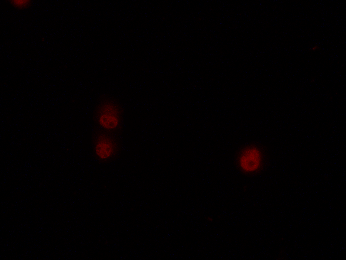


Sophocarpine
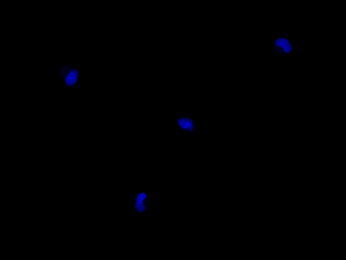

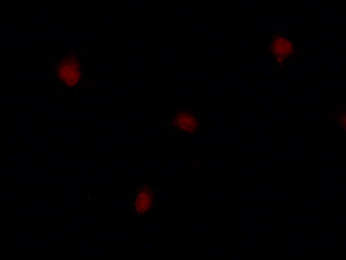


**PC3 (Collagen I)**

Control
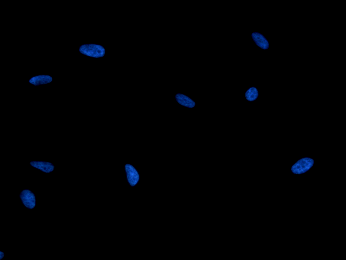

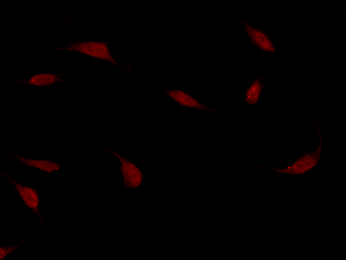


Sophocarpine
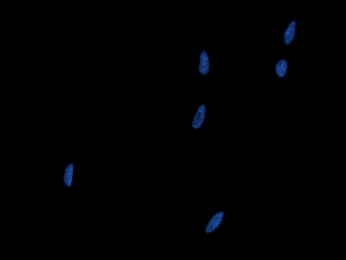

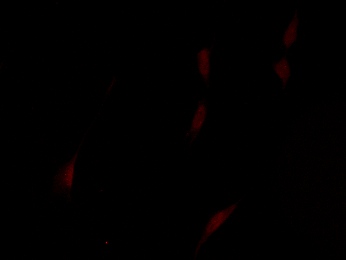


**Figure 5**

**（A）Western Blotting**

**DU145**

PI3K
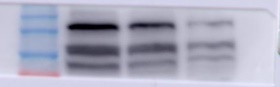


AKT
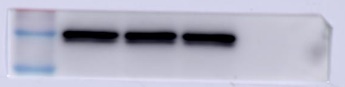


p-AKT
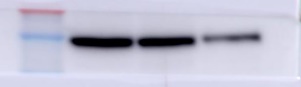


mTOR
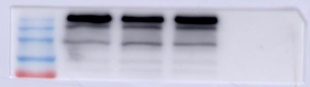


p-mTOR
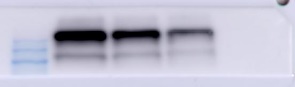


GAPDH
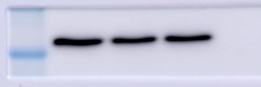


**PC3**

PI3K
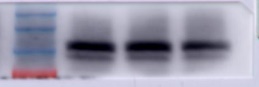


AKT
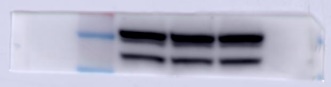


p-AKT
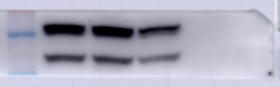


mTOR
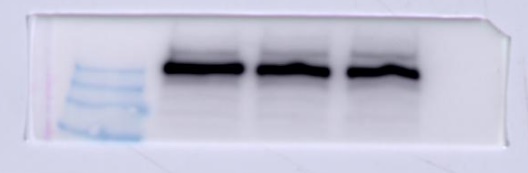


p-mTOR
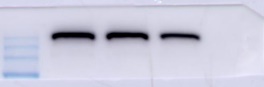


GAPDH
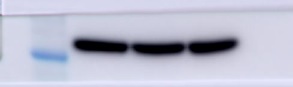


**(B) Immunofluorescence Analysis**

**DU145 (p-mTOR)**

Control
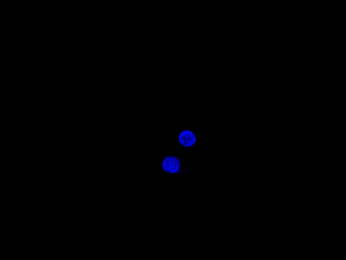

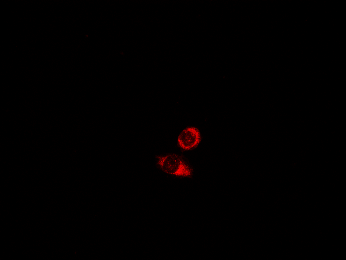


Sophocarpine
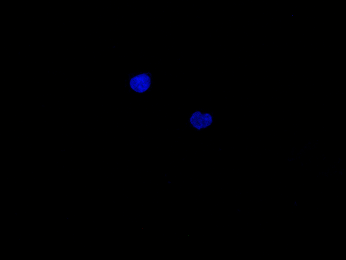

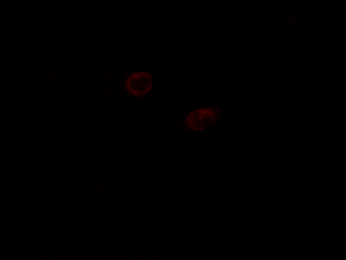


**PC3 (p-mTOR)**

Control
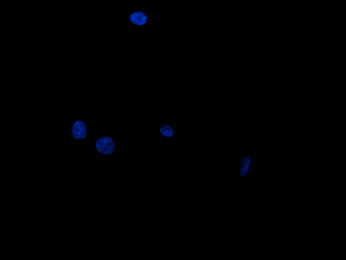

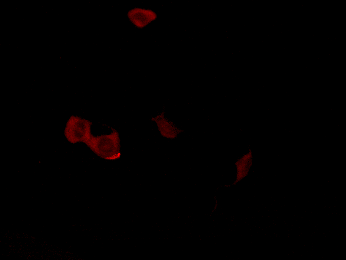


Sophocarpine
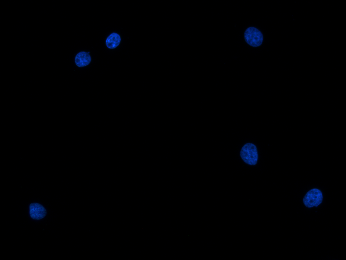

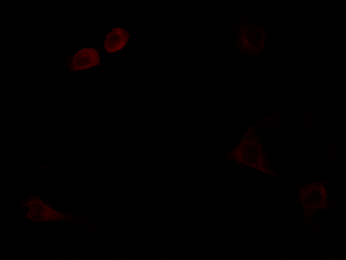


**(C) Molecular docking**

**
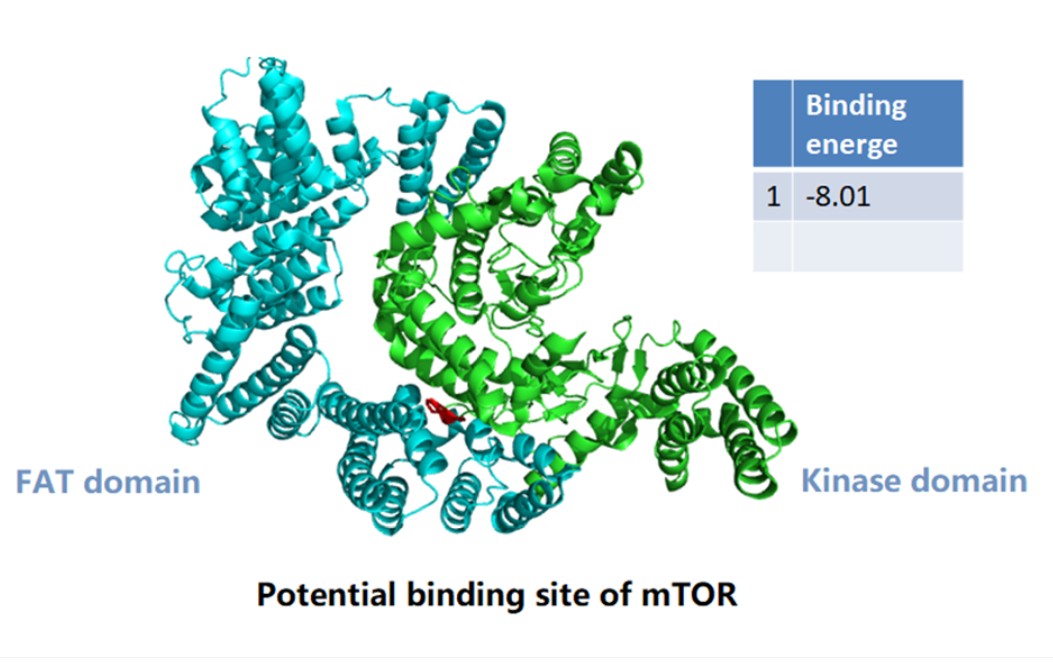

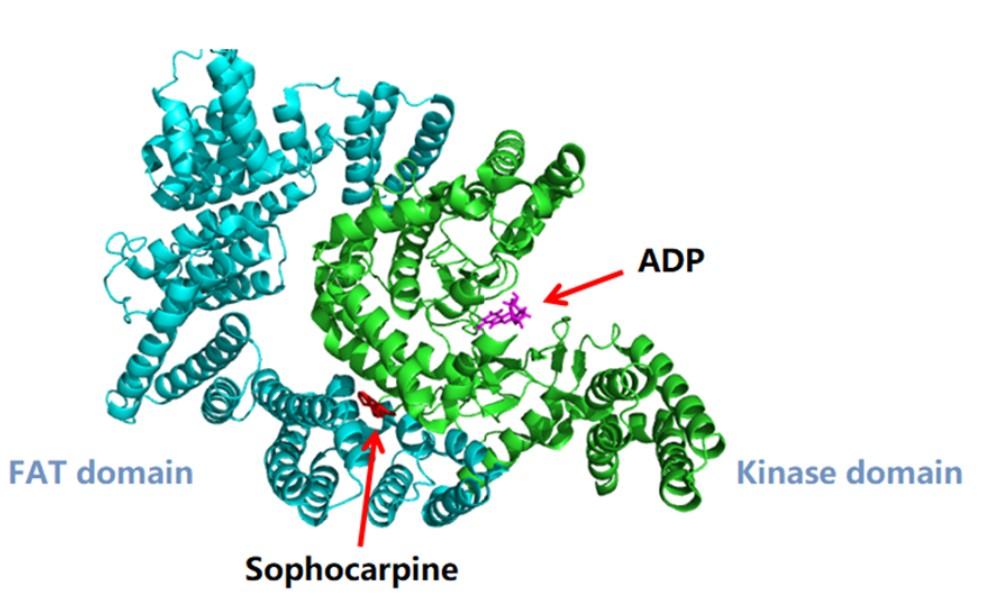
**

**Figure 6**

**(A) Appearance of tumor**

**
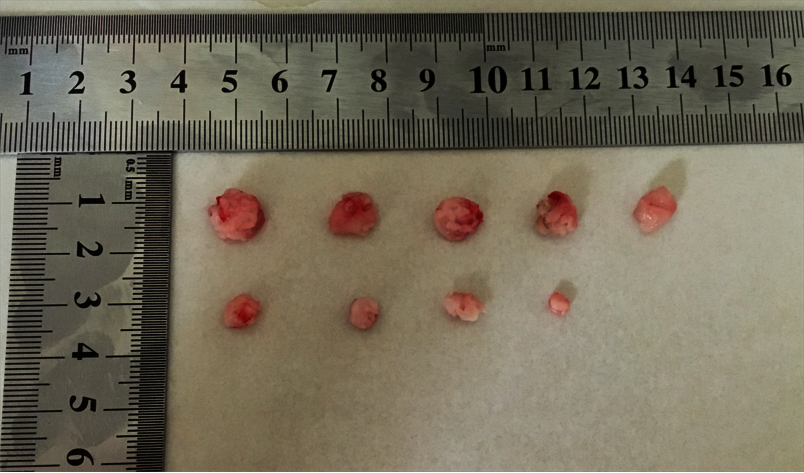
**

**(D) Western Blotting**

**Tumor**

PI3K
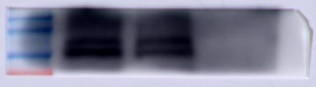


AKT
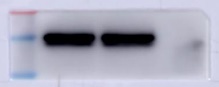


p-AKT
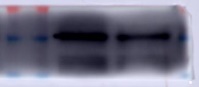


mTOR
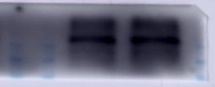


p-mTOR
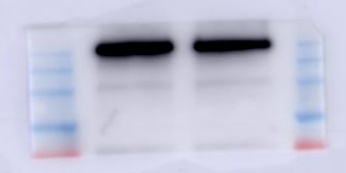


GAPDH
